# Reabilitação Cardíaca Baseada em Exercícios Fortemente Relacionada com Redução do Volume Plaquetário Médio

**DOI:** 10.36660/abc.20190514

**Published:** 2021-02-03

**Authors:** İsmet Durmuş, Ezgi Kalaycıoğlu, Mustafa Çetin, Hanife Baykal Şahin, Tuncay Kırış

**Affiliations:** 1 University of Health Sciences Turkey Ahi Evren Chest and Cardiovascular Surgery Education Research Hospital Trabzon Turquia University of Health Sciences Turkey , Ahi Evren Chest and Cardiovascular Surgery Education and Research Hospital , Trabzon - Turquia; 2 Recep Tayyip Erdogan University, Faculty of Medicine Rize Turquia Recep Tayyip Erdogan University, Faculty of Medicine , Rize – Turquia; 3 zmir Katip Çelebi University Atatürk Training and Research Hospital Department of Cardiology İzmir Turquia zmir Katip Çelebi University Atatürk Training and Research Hospital , Department of Cardiology , İzmir - Turquia

**Keywords:** Reabilitação Cardíaca, Exercício, Atividade Física, Volume Plaquetário Médio, Plaquetas, Doença da Artéria Coronariana, Prognóstico, Ecocardiografia/métodos

## Abstract

**Fundamento:**

O volume plaquetário médio (VPM), uma medida simples de ativação plaquetária, tornou-se recentemente um tópico interessante no campo da pesquisa cardiovascular. A reabilitação cardíaca (RC) baseada em exercícios é uma intervenção abrangente que diminui a morbidade-mortalidade em pacientes com doença arterial coronariana (DAC). Estudos sobre os efeitos do exercício físico na ativação plaquetária têm produzido resultados conflitantes.

**Objetivo:**

O objetivo deste estudo foi determinar o efeito de um programa de RC baseado em exercícios sobre o VPM em pacientes com DAC estável.

**Métodos:**

A amostra foi composta por 300 pacientes consecutivos com DAC estável. Os pacientes foram divididos em dois grupos: grupo RC (n = 97) e grupo não RC (n = 203). Foi feito um hemograma. As medidas de correlação ponto-bisserial foram tiradas para mostrar a correlação entre a alteração do VPM e a RC. Valor de p<0,05 foi considerado estatisticamente significativo.

**Resultados:**

A diminuição do VPM foi maior no grupo CR do que no grupo não CR [(-1,10 (-1,40-(-0,90)) vs. (-0,10 (-2,00-0,00)); p<0,001]. ΔVPM teve correlação positiva com Δ neutrófilos (r = 0,326, p<0,001), ΔTG (r = 0,439, p<0,001), ΔLDL-c (r = 0,478, p<0,001), ΔGB (r = 0,412, p<0,001) e ΔPCR (r = 0,572, p <0,001). Foi encontrada uma correlação significativa entre ΔVPM% e CR (r = 0,750, p <0,001).

**Conclusões:**

Pudemos mostrar que a RC baseada em exercícios tem forte relação com a redução do VPM em pacientes com DAC. Consideramos que a diminuição da ativação plaquetária com RC baseada em exercícios pode desempenhar um papel importante na redução do risco trombótico em pacientes com DAC estável. (Arq Bras Cardiol. 2020; [online].ahead print, PP.0-0)

## Introdução

A doença cardiovascular (CV) é uma das principais causas de mortalidade e incapacidade e continua sendo uma grande preocupação, apesar da melhora dos resultados clínicos com o tratamento baseado em evidências. ^1 , 2^ As plaquetas são essenciais para a hemostasia primária e reparo do endotélio, mas também desempenham um papel fundamental na patogênese da aterosclerose e trombose arterial. ^1^ A ativação plaquetária está associada a eventos cardiovasculares. ^3^ O monitoramento da função das plaquetas pode ajudar na avaliação do prognóstico de pacientes com doença arterial coronariana (DAC). ^4^

No entanto, como o teste de função plaquetária é demorado, caro e tecnicamente desafiador, não é amplamente utilizado. ^4^ Em comparação com plaquetas menores, as plaquetas maiores contêm grânulos densos, expressam mais receptores de adesão e induzem maior atividade trombótica, podendo ser um reflexo do grau de ativação plaquetária. ^5^ O volume plaquetário médio (VPM) é um parâmetro importante de identificação do tamanho das plaquetas que foi proposto como indicador da reatividade plaquetária e é normalmente determinado por analisadores de contagem completa a um custo relativamente baixo. ^6^ Níveis elevados de VPM têm sido associados a DAC, infarto do miocárdio, doença arterial periférica, doença cerebrovascular e desfecho desfavorável. ^7^

A reabilitação cardíaca (CR) baseada em exercícios é uma intervenção abrangente que inclui treinamento físico supervisionado, gerenciamento de fatores de risco, orientação do paciente e aconselhamento psicossocial. ^8^ A CR tem se mostrado eficaz na melhora de sintomas isquêmicos de esforço, tolerância ao exercício e fatores de risco coronariano em pacientes com DAC. Além disso, demonstrou reduzir a mortalidade por todas as causas e por eventos cardiovasculares em 20% a 32% entre os pacientes com DAC. ^9^

Estudos sobre os efeitos do exercício na ativação plaquetária têm produzido resultados conflitantes. ^2^ O objetivo deste estudo foi determinar o efeito de um programa de RC baseado em exercícios no VPM em pacientes com DAC estável.

## Métodos

### População do estudo

O tamanho da amostra foi determinado com poder de 80% e margem de erro de 5% após avaliação preliminar de 5 a 10 casos. O estudo incluiu 300 pacientes ambulatoriais consecutivos que foram submetidos a angiografia coronária (CAG) nos seis meses anteriores devido a angina de peito estável e >50% de estenose detectada em pelo menos uma artéria coronária, ou tinham histórico de intervenção coronária percutânea (ICP)/cirurgia coronária de revascularização do miocárdio (CRM), e foi encaminhado para um programa de RC de fase III. O grupo não RC foi composto por 97 pacientes que não concordaram em participar do programa de reabilitação. Foram excluídos pacientes com doenças imunológicas ou inflamatórias, doenças hematológicas, sepse, infecções locais ou sistêmicas ativas, doença renal crônica (eTFG <30Ml/min/1,73m ^2^ ), idade ≤18 e >80, fração de ejeção do ventrículo esquerdo (FEVE) <40%, ou com história de malignidade.

Os tratamentos dos pacientes foram otimizados antes da participação, e nenhum deles teve alterações na medicação durante o estudo.

Foi realizado o hemograma nos pacientes incluídos no grupo RC um dia antes do início do programa e um dia após o término do programa (que durou seis semanas), após jejum de 12 horas. Por outro lado, nos pacientes que não foram incluídos no programa, foi coletado sangue para hemograma no momento de inclusão no estudo e seis semanas depois, em jejum de 12 horas. Todas as amostras foram obtidas em tubos padronizados de ácido etilenodiamino tetra-acético (EDTA). O hemograma foi medido em analisador hematológico automatizado Advia 2120 (Siemens). Glicose em jejum, colesterol LDL (LDC-c), triglicerídeos (TG), colesterol HDL (HDL-c), glóbulos brancos (GB), proteína C reativa (PCR), creatinina e hemoglobina foram medidos. O índice de massa corporal (IMC) foi calculado como peso (kg)/altura (m ^2^ ).

O cálculo do escore de Gensini foi iniciado atribuindo-se um escore de gravidade para cada estenose coronária: 1 ponto para estreitamento ≤25%, 2 pontos para estreitamento de 26 a 50%, 4 pontos para estreitamento de 51 a 75%, 8 pontos para estreitamento de 76 a 90% estreitamento, 16 pontos para estreitamento de 91 a 99% e 32 pontos para oclusão total. A partir daí, cada pontuação de lesão foi multiplicada por um fator que leva em consideração a importância da posição da lesão na circulação coronária (5 para o tronco da coronária esquerda, 2,5 para o segmento proximal da coronária descendente anterior esquerda, 2,5 para a proximal segmento da artéria circunflexa, 1,5 para o segmento médio da artéria coronária descendente anterior esquerda, 1,0 para a artéria coronária direita, o segmento distal da artéria coronária descendente anterior esquerda, a artéria póstero-lateral e a artéria marginal obtusa, e 0,5 para outros segmentos). Finalmente, o escore de Gensini foi calculado pela soma das pontuações dos segmentos coronários individuais. ^10^

O Ecocardiograma foi feito com base nas diretrizes da Sociedade Americana de Ecocardiografia. O volume diastólico final (VDF) e o volume sistólico final (VSF) do ventrículo esquerdo (VE) foram calculados a partir da visualização do corte apical de duas e quatro câmaras usando o método de Simpson modificado. A FEVE foi calculada como FEVE=(VDF-VSF)/VSFX100.

O estudo foi realizado nos termos da Declaração de Helsinque e com a aprovação do conselho de ética local.

### Programa de RC

Um teste incremental em cicloergômetro foi aplicado antes do programa de RC em pacientes do grupo RC, para determinar a capacidade de exercício. Durante o teste, o objetivo era atingir a frequência cardíaca máxima esperada de acordo com a idade (220 menos a idade). As indicações para encerrar o teste de esforço foram: desvio do segmento ST, angina moderada a grave, queda na pressão arterial sistólica >10 mmHg (persistentemente abaixo da linha de base), apesar de um aumento na carga de esforço, resposta hipertensiva (pressão arterial sistólica >250 mmHg e/ou pressão arterial diastólica >115 mmHg), sintomas do sistema nervoso central (por exemplo, ataxia, tontura ou quase síncope), fadiga, falta de ar, respiração ofegante, cãibras nas pernas ou claudicação. Após descanso de dois minutos, a carga de esforço foi aumentada em 25W a cada dois minutos. A frequência cardíaca e a pressão arterial foram monitoradas durante todo o teste. A carga máxima foi determinada pela capacidade máxima de exercício.

O programa de RC foi realizado com a supervisão de uma equipe multidisciplinar, incluindo um cardiologista, um fisioterapeuta experiente como coordenador e um especialista em fisioterapia e reabilitação como diretor médico. O programa foi realizado no centro de reabilitação cardíaca do nosso hospital de cardiologia e cirurgia cardiovascular.

O programa de RC consiste em exercícios aeróbicos e exercícios de relaxamento. Com base no resultado do teste ergométrico, a prescrição do exercício foi agendada individualmente. Os pacientes permaneceram no programa cinco dias por semana, por um total de seis semanas. Todos os pacientes do grupo RC concluíram o programa. Cada sessão durava 30 minutos, incluindo o aquecimento de cinco minutos e o relaxamento final de cinco minutos. A intensidade do exercício aeróbio foi prescrita de acordo com a capacidade de exercício de cada indivíduo.

A intensidade começou em 40-50% da reserva de frequência cardíaca máxima e gradualmente aumentou para 70-85% da reserva de frequência cardíaca máxima. A reserva de frequência cardíaca foi avaliada pela fórmula de Karvonen (FCtreino = (FCmáx. – FCrepouso) x Intensidade do exercício + FCrepouso). ^11^ FCtreino sendo a frequência cardíaca durante o exercício aeróbio, FCmáx a frequência cardíaca máxima alcançada no teste de cicloergômetro, e FCrepouso a frequência cardíaca em repouso. A Escala de Taxa de Percepção de Esforço (RPE, *Rate of Perceived Exertion* ) de Borg foi usada, e os pacientes se exercitaram com RPE de 13-15. Os pacientes foram monitorados continuamente por eletrocardiografia (ECG) com um transmissor de ECG de 1 canal (Custo med, Ottobrunn, Alemanha) e as medições de pressão arterial sistólica/diastólica eram realizadas automaticamente a cada cinco minutos por meio de um sistema de software (Custo med, Ottobrunn, Alemanha). Durante o estudo, os pacientes também foram encaminhados a um psicólogo, um nutricionista e uma clínica para parar de fumar.

### Análise estatística

As análises estatísticas foram realizadas no software estatístico SPSS (versão 21.0, SPSS, Chicago, IL, EUA). Variáveis contínuas são apresentadas em média e desvio-padrão. Variáveis categóricas são apresentadas como números e porcentagens. As variáveis foram comparadas usando o teste t de Student bicaudal para variáveis contínuas de distribuição normal ou o teste U de Mann-Whitney para variáveis contínuas de distribuição não normal. O teste de Kolmogorov-Smirnov foi aplicado para verificar a normalidade da distribuição das variáveis contínuas, e o teste do qui-quadrado para variáveis categóricas. O teste relacionado ou teste dos postos sinalizados de Wilcoxon foi usado para comparar as variáveis antes e depois da terapia. A correlação de Spearman foi realizada para examinar a relação entre as variáveis contínuas. Medidas de correlação ponto-bisserial foram realizadas para mostrar a correlação entre as alterações do VPM e a RC. Valor de p<0,05 foi adotado como estatisticamente significativo.

## Resultados

A população do estudo (300 pacientes) foi dividida em dois grupos de acordo com seu ingresso no programa de RC. Duzentos e três pacientes participaram do programa de RC (grupo RC) e 97 pacientes, não (grupo não RC). Características demográficas e clínicas, bem como achados laboratoriais da população, estão listados na Tabela 1 . Histórico de síndrome coronariana aguda, CRM e ICP foi semelhante nos dois grupos ( Tabela 1 ).

**Tabela 1:** – Características clínicas e laboratoriais dos grupos RC e não-RC

Variáveis	Grupo não-RC (n=97)	Grupo RC (n=203)	Valor de p
Idade (anos)	57,9±7,4	56,2±7,8	0,072
Sexo (masc. %)	74(76,3%)	159(78,3%)	0,399
Hipertensão	82(84,5%)	184(90,6%)	0,088
Diabetes mellitus (n, %)	20(20,6%)	55(27,1%)	0,142
Fumante (n, %)	11(11,3%)	27(13,3%)	0,391
IMC (kg/m ^2^ )	28,2±3,1	28,3±3,9	0,885
Histórico de ICP (n, %)	61(62,9%)	117(57,6%)	0,230
Histórico de SCA (n, %)	28(28,9%)	50(24,6%)	0,259
Histórico de CRM (n, %)	19(19,6%)	37(18,2%)	0,446
LVEF %	57,1±5,3	58,3±5,6	0,086
Gravidade da DAC			
1 vaso	35(36,1%)	89(44,1%)	
2 vasos	58(59,8%)	98(48,5%)	
>2 vasos	4(4,1%)	15(7,4%)	0,155
Score de Gensini	54,1±28	50,1±28,2	0,266
AAS (n, %)	95(97,9%)	200(98,5%)	0,745
Inibidores de P2Y12 (n, %)	60(61,5%)	115(56,6%)	0,493
Bloqueadores beta (n, %)	82(84,5%)	158(77,8%)	0,113
Bloqueadores dos canais de cálcio (n, %)	42(43,3%)	91(44,8%)	0,451
Inibidores RAAS (n, %)	80(82,5%)	165(81,3%)	0,469
Estatina (n, %)	92(94,8%)	197(97,5%)	0,377
Glicose plasmática em jejum (pré) (mg/dL)	102,6±27,1	106,2±33,7	0,307
Creatinina (pré) (mg/dl)	0,9±0,21	0,87±0,21	0,374
Hemoglobina (pré) (g/dL)	13,4±1,02	13,6±1,07	0,135
VPM (pré)	8,7±0,98	9,1±1,02	0,010
VPM (pós)	8,6±0,98	7,9±0,82	<0,001
ΔVPM *	-0,10 (-2,00-0,00)	-1,10(-1,40-[-0,90])	<0,001*
ΔVPM %*	-1,15 (-2,37-0,00)	-12,63(-14,46-[-10,11])	<0,001*
ΔLDL-c (mg/dL)*	-2,00 (-1,00-0,00)	-20,00 (-36,00-[-10,00])	<0,001*
ΔTG (mg/dL)*	-9,00 (-10,50-[-1,00])	-21,00 (-49,00-[-10,00])	<0,001*
ΔHDL-c (mg/dL)*	1,00 (0,00-1,00)	4,00 (3,00-6,00)	<0,001*
ΔGB (10 ^3^ /µ ^3^ )*	0,10 (-0,25-0,30)	-0,70(-1,20-[-0,35])	<0,001*
ΔNeutrófilos (10 ^3^ /µ ^3^ ) *	-0,10 (-0,50-0,03)	-0,80 (-1,50-[-0,39])	<0,001*
ΔPCR (mg/dL) *	-0,10 (-0,10-0,10)	-0,80 (-1,80-[-0,40])	<0,001*

Dados apresentados como média ± desvio-padrão ou número de pacientes e porcentagem de pacientes. CR: reabilitação cardíaca; Δ: delta; IMC: índice de massa corporal; ICP: intervenção coronária percutânea; SCA; síndrome coronariana aguda; CRM: cirurgia de revascularização do miocárdio; FEVE: fração de ejeção do ventrículo esquerdo; DAC: doença arterial coronariana; AAS: ácido acetilsalicílico; RAAS: renina-angiotensina-aldosterona; VPM: volume plaquetário médio; LDL: lipoproteína de baixa densidade; TG: Triglicerídeos; HDL: lipoproteína de alta densidade; GB: glóbulo branco; PCR: proteína C-reativa; pré: antes da RC. *Comparação pelo teste U de Mann-Whitney em p<0,05. Os valores foram descritos como medianas com variação interquartil (percentis 25º e 75º).

**Figura 1:**
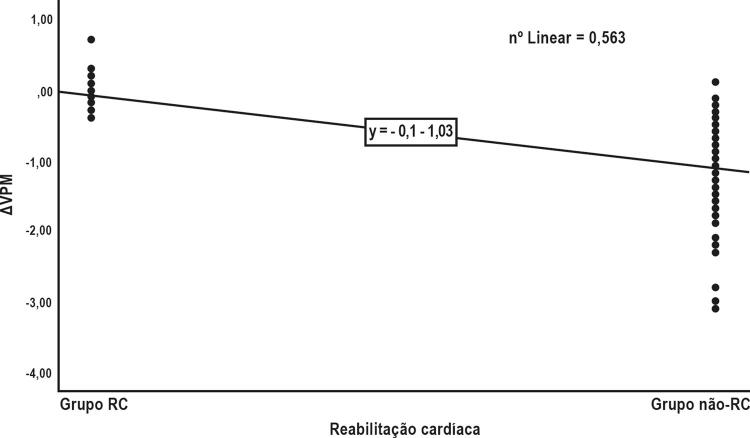
Correlação do ΔVPM com CR.

A diminuição no VPM foi maior no grupo RC do que no grupo não RC [((-1,10 (-1,40 - (-0,90)) vs. (-0,10 (-2,00-0,00)) vs.; p<0,001, Tabela 1 ]. A correlação entre ΔMPV e as variáveis é mostrada na Tabela 2 . Conforme mostrado na Figura 1 , o ΔMPV teve forte correlação positiva com a RC (r = 0,750, p <0,001).

**Tabela 2:** – Correlação entre ΔVPM e variáveis

	ΔVPM	
	r	valor de p
ΔNeutrófilos	0,326	<0,001
ΔLDL-c	0,478	<0,001
ΔGB	0,412	<0,001
ΔPCR	0,572	<0,001
ΔTG	0,439	<0,001
FEVE	-0,133	0,021
hemoglobina (pré)	-0,139	0,019
Glicose plasmática em jejum (pré)	-0,129	0,026
ΔHDL-c	-0,537	<0,001
RC	0,750	<0,001
Idade	0,034	0,563
IMC	-0,022	0,727

*Δ: delta; VPM: volume plaquetário médio; LDL: lipoproteína de baixa densidade; TG: Triglicerídeos; FEVE: fração de ejeção do ventrículo esquerdo; HDL: lipoproteína de alta densidade; GB: glóbulo branco; PCR: proteína C-reativa; RC: reabilitação cardíaca; IMC: índice de massa corporal; pré: antes de CR.*

## Discussão

Este estudo mostrou que a RC baseada em exercícios tem uma forte relação com a redução do VPM em pacientes com DAC.

Estudos sobre o efeito do exercício na ativação plaquetária têm produzido resultados conflitantes até o momento. Em concordância com nosso estudo, Yazıcıet al. ^7^ relataram que uma mudança no estilo de vida que incluísse pelo menos 180 minutos/semana de atividades físicas de intensidade moderada diminuiu o VPM em pacientes pré-hipertensos. Além disso, Rauramaa et al., ^12^ mostraram que o treinamento físico de intensidade baixa a moderada se associou à diminuição da agregação plaquetária. Contrário a esses resultados, também foi demonstrado que o teste ergométrico aumentou o VPM em pacientes com DAC. ^11 , 13^ Além disso, há registros de que exercícios resistidos graduais aumentam o VPM. ^12 , 14^

O treinamento físico pode trazer efeitos benéficos às plaquetas por meio de diferentes mecanismos. Com o treinamento físico, o fluxo pulsátil na aorta aumenta, e isso pode induzir uma liberação aguda e suprarregulação de óxido nítrico (NO), que é um mediador potente dos efeitos antiplaquetários e supressão da reatividade plaquetária. ^7 , 15^ Sabe-se que a RC aumenta o colesterol HDL, o que pode estimular a produção de plaquetas e, assim, diminuir a ativação plaquetária. ^9^ Em nosso estudo, o colesterol ΔHDL foi correlacionado de forma independente com a diminuição do VPM.

O VPM é reconhecido como um marcador inflamatório em doenças cardiovasculares, cerebrovasculares, reumatológicas e gastroenterológicas. ^16^ Recentemente, demosntrou-se que a presença de megacariócitos ativados na medula óssea se correlaciona com o aumento dos níveis circulantes de IL-6 em pacientes com aterosclerose. ^17^ Pesquisas anteriores forneceram evidências de que um estilo de vida fisicamente ativo está associado a biomarcadores inflamatórios inferiores em todo o corpo, e as ações anti-inflamatórias do treinamento físico crônico são evidentes após 2 a 12 semanas de treinamento supervisionado. ^18^ Em nosso estudo, demonstramos que ΔPCR foi um dos preditores independentes de ΔMPV. A redução do VPM após a RC pode ser explicada pelo efeito anti-inflamatório dos programas de RC baseados em exercícios.

As contradições nos resultados dos estudos podem ser explicadas por vários aspectos do exercício que afetam as funções das plaquetas, como diferentes intensidades de exercício, durações e vários níveis de aptidão dos indivíduos. ^7^ Está comprovado que o exercício intenso agudo aumenta as citocinas pró-inflamatórias plasmáticas. ^18^ O VPM pode refletir as mudanças na estimulação plaquetária e no nível de produção de plaquetas. ^13^ Um aumento no VPM após o exercício pode ser atribuído à liberação recente de plaquetas grandes jovens, particularmente do pool esplênico, para a circulação. ^7^ Neste estudo, implementamos o programa de RC cinco dias por semana por um total de seis semanas com densidade gradativa crescente, de acordo com a capacidade de exercício de cada indivíduo.

A aterosclerose em si pode estimular megacariócitos da medula óssea, que se mostrou associada à síndrome coronariana aguda, por induzir consumo de plaquetas circulantes durante a aterogênese. ^17 - 19^ Um dos mecanismos da RC baseada em exercícios potencialmente envolvido na redução de infarto e reinfarto do miocárdio pode estar relacionado com a redução do VPM. ^9 , 20^

As plaquetas reticuladas são maiores e possivelmente mais ativas do que as plaquetas não reticuladas. ^1^ Além disso, as reticuladas ou grandes exibem reatividade aumentada apesar da terapia antiplaquetária. ^21 , 22^ Uma vez que foi demonstrado que o VPM alto é um fator de risco independente para infarto do miocárdio (IM) futuro e recorrente, estando associados à síndrome coronariana aguda ou a fatores de risco cardiovascular, ^1 , 19 , 23^ levantou-se o questionamento sobre como diminuir o VPM. ^17 , 23^ Embora tenha sido demonstrado anteriormente que o tratamento com estatinas diminui o VPM, ^21^ em nosso estudo a maioria dos pacientes já estava em uso de estatinas, então nos ativemos ao efeito aditivo da RC na redução do VPM.

### Limitações do estudo

O presente estudo teve algumas limitações. Primeiro, avaliamos o VPM apenas uma vez e não checamos as mudanças nele ao longo do tempo. Em segundo lugar, alguns medicamentos antiplaquetários podem afetar o tamanho das plaquetas.

## Conclusão

Este estudo mostrou que a RC baseada em exercícios tem forte relação com a redução do VPM em pacientes com DAC. Consideramos que a diminuição da ativação plaquetária com RC baseada em exercícios pode desempenhar um papel importante na redução do risco trombótico em pacientes com DAC estável.
