# Supplementary material for: Reabilitação Cardíaca Baseada em Exercícios Fortemente Relacionada com Redução do Volume Plaquetário Médio
Source: Arq Bras Cardiol. 2021 Feb 3;116(3):434–40. [Article in Portuguese] doi: 10.36660/abc.20190514 (PMC8159561; doi:10.36660/abc.20190514)
Supplement: Translation

# Translation: Exercise-Based Cardiac Rehabilitation Has a Strong Relationship with Mean Platelet Volume Reduction

**Authors:** İsmet Durmuş, Ezgi Kalaycıoğlu, Mustafa Çetin, Hanife Baykal Şahin, Tuncay Kırış

**Affiliations:** 1 Turkey University of Health Sciences Turkey, Ahi Evren Chest and Cardiovascular Surgery Education and Research Hospital, Trabzon - Turkey; 2 Turkey Recep Tayyip Erdogan University, Faculty of Medicine, Rize – Turkey; 3 Turkey zmir Katip Çelebi University Atatürk Training and Research Hospital, Department of Cardiology, İzmir - Turkey

**Keywords:** Cardiac, Rehabilitation, Exercise, Physical, Activity, Mean Platelet Volume, Blood Platelets, Coronary artery Disease, Echocardiography/methods

## Abstract

**Background:**

Mean platelet volume (MPV), which is a simple measure of platelet activation, has recently become an interesting topic in cardiovascular research. Exercise-based cardiac rehabilitation (CR) is a comprehensive intervention that decreases mortality-morbidity in patients with coronary artery disease (CAD). Studies on the effects of exercise on platelet activation have yielded conflicting results.

**Objective:**

The purpose of this study was to determine the effect of an exercise-based CR programs on MPV in patients with stable CAD.

**Methods:**

The sample was composed of 300 consecutive stable CAD patients. The patients were divided into two groups: CR group (n = 97) and non-CR group (n = 203). Blood analysis was performed. Point-Biserial correlation measures were performed to show correlation between MPV change and CR. A p value of <0.05 was considered statistically significant.

**Results:**

The decrease in MPV was greater in the CR group than in the non-CR group [(-1.10(-1.40-(-0.90)) vs. (-0.10 (-2.00-0.00)); p< 0.001]. ΔMPV had a positive correlation with Δ neutrophil (r = 0.326, p < 0.001), ΔTG (r = 0.439, p < 0.001), ΔLDL-c (r = 0.478, p < 0.001), ΔWBC (r = 0.412, p < 0.001), and ΔCRP (r = 0.572, p < 0.001). A significant correlation was found between ΔMPV% and CR (r=0.750, p<0.001).

**Conclusions:**

We were able to show that exercise-based CR has a strong relationship with MPV reduction in patients with CAD. We consider that decreased platelet activation with exercise-based CR might play an important role in reducing thrombotic risk in patients with stable CAD. (Arq Bras Cardiol. 2020; [online].ahead print, PP.0-0)

## Introduction

Cardiovascular (CV) disease is one of the leading causes of mortality and disability and remains a major concern despite improved clinical outcomes with evidence-based treatment.^1 , 2^Platelets are essential for primary hemostasis and repair of the endothelium, but they also play a key role in the pathogenesis of atherosclerosis and arterial thrombosis.^1^Platelet activation is associated with CV events.^3^Monitoring the function of platelets may help to evaluate the prognosis of patients with coronary artery disease (CAD).^4^

However, because platelet function testing is a time-consuming, costly, and technically challenging process, it is not widely used.^4^Compared with smaller platelets, larger platelets contain dense granules, express more adhesion receptors, and induce higher thrombotic activity, which can reflect the degree of platelet activation.^5^Mean platelet volume (MPV) is a major parameter of platelet size that has been proposed as an indicator of platelet reactivity and is routinely determined by complete count analyzers at a relatively low cost.^6^Increased MPV levels have been reported to be associated with CAD, myocardial infarction, peripheral arterial disease, cerebrovascular disease, and poor outcome.^7^

Exercise-based cardiac rehabilitation (CR) is a comprehensive intervention that includes medically supervised exercise training, risk factor management, patient education, and psychosocial counseling.^8^CR has been proven effective in improving exertional ischemic symptoms, exercise tolerance, and coronary risk factors in patients with CAD. Also, it was shown to also reduce all-cause and CV mortality in 20% to 32% among patients with CAD.^9^

Studies on the effects of exercise on platelet activation have yielded conflicting results.^2^The purpose of this study was to determine the effect of an exercise-based CR program on MPV in patients with stable CAD.

## Methods

### Study population

Sample size was determined with 80% power and 5% margin of error after preliminary evaluation of 5-10 cases. The study included 300 consecutive outpatients who had undergone coronary angiography (CAG) in the previous six months due to stable angina pectoris and >50% stenosis detected in at least one coronary artery, or had a history of percutaneous coronary intervention (PCI)/coronary artery bypass graft (CABG) surgery, and had been referred to a Phase-III CR program. The non-CR group was composed of 97 patients who did not agree to participate in the CR program. Patients with immunologic or inflammatory diseases, hematological diseases, sepsis, active local or systemic infections, chronic renal disease (eGFR<30Ml/min/1.73m^2^), age ≤18 and >80, left ventricular ejection fraction (LVEF) <40%, or with history of malignancy were excluded.

Patients’ medical treatments were optimized before the participation, and none of them had changes in medication during the study.

Blood analysis was performed in patients included in the CR group one day before the start of the program and one day after the end of the program (which lasted six weeks) after a 12-hour fasting. On the other hand, in patients who were not included in the CR program, blood analysis was performed upon their inclusion in the study and six weeks later, after a 12-hour fasting. All samples were obtained in standardized dipotassium ethylenediaminetetraacetic acid (EDTA) tubes. The blood counts were measured using an automated hematology analyzer Advia 2120 (Siemens). Fasting blood glucose, low-density lipoprotein cholesterol (LDC-c), triglycerides (TG), high-density lipoprotein cholesterol (HDL-c), white blood cell (WBC), C-reactive protein (CRP), creatinine, and hemoglobin values were measured. Body mass index (BMI) was calculated as weight (kg)/height (m^2^).

Calculation of the Gensini score was initiated by giving a severity score to each coronary stenosis: 1 point for ≤25% narrowing, 2 points for 26 to 50% narrowing, 4 points for 51 to 75% narrowing, 8 points for 76 to 90% narrowing, 16 points for 91 to 99% narrowing, and 32 points for total occlusion. Thereafter, each lesion score was multiplied by a factor that takes into account the importance of the lesion’s position in the coronary circulation (5 for the left main coronary artery, 2.5 for the proximal segment of the left anterior descending coronary artery, 2.5 for the proximal segment of the circumflex artery, 1.5 for the mid-segment of the left anterior descending coronary artery, 1.0 for the right coronary artery, the distal segment of the left anterior descending coronary artery, the poster lateral artery, and the obtuse marginal artery, and 0.5 for other segments). Finally, the Gensini score was calculated by summation of the individual coronary segment scores.^10^

Echocardiographic measurements were taken according to the American Society of Echocardiography guidelines. The left ventricle (LV) end-diastolic volume (EDV) and end-systolic volume (ESV) were calculated from the apical two- and four-chamber views using the modified Simpson’ method. The LVEF was calculated as LVEF= (EDV-ESV)/EDVX100.

This study was performed in accordance with the Declaration of Helsinki and with the approval by the local ethics board.

### CR program

A step incremental cycle ergometer test was applied before the CR program in patients in the CR group, so as to determine their exercise capacity. During the test, the aim was to reach the expected maximum heart rate according to age (220 minus age). The indications for terminating the exercise testing were: ST-segment deviation, moderate to severe angina, drop in systolic blood pressure >10 mmHg (persistently below baseline) despite an increase in workload, hypertensive response (systolic blood pressure >250 mmHg and/or diastolic blood pressure >115 mmHg), central nervous system symptoms (e.g., ataxia, dizziness, or near syncope), fatigue, shortness of breath, wheezing, leg cramps, or claudication. After a two-minute resting period, the workload was increased by 25W every two minutes. Heart rate and blood pressure were measured during the whole test. The maximum workload was determined as the maximal exercise capacity.

The CR program was performed with the supervision of a multidisciplinary team, including a cardiologist, an experienced physical therapist as a coordinator, and a physical therapy and rehabilitation specialist as the medical director. The rehabilitation program was performed in the CR center of our cardiology and cardiovascular surgery hospital.

The CR program consists of aerobic exercise training and relaxation exercises. Based on the result of the exercise testing, exercise prescription was scheduled individually. Patients remained in the program five days a week, for a total of six weeks. All patients in the CR group completed the program. Each session lasted 30 minutes, including the five-minute warm-up and the final five-minute cool-down. The aerobic exercise intensity was prescribed according to the individual’s exercise capacity. The intensity began at 40-50% of maximal heart rate reserve and gradually increased to 70-85% of maximal heart rate reserve. Heart rate reserve was evaluated by the Karvonen Formula (HRtrain = (HRmax – HRrest) x ExerciseIntensity + HRrest).^11^HRtrain being the heart rate during the aerobic exercise, HRmax the maximum heart rate reached thorough the cycle ergometer test, and HRrest the heart rate at rest. The Borg Scale of Rate of Perceived Exertion (RPE) was used, and patients exercised at an RPE of 13-15. Patients were continuously monitored by electrocardiography (ECG) with a 1-channel ECG transmitter (Custo med, Ottobrunn, Germany), and systolic/diastolic blood pressure measurements were performed automatically every five minutes via a software system (Custo med, Ottobrunn, Germany). During the study, patients were also directed to a psychologist, a dietitian, and a smoking cessation clinic.

### Statistical analysis

Statistical analyses were carried out using the SPSS statistical software (version 21.0, SPSS, Chicago, IL, USA). Continuous variables are presented as mean and standard deviation. Categorical variables are presented as numbers and percentages. The variables were compared using the two-tailed student t test for continuous variables of normal distribution or the Mann-Whitney U test for continuous variables of non-normal distribution. The Kolmogorov-Smirnov test was applied to verify the normality of the distribution of continuous variables. The chi-square test was used for categorical variables. The related test or Wilcoxon signed-rank test was used to compare variables before and after therapy. Spearman’s correlation analysis was performed to examine the relationship between continuous variables. Point-Biserial correlation measures were performed to show the correlation between MPV changes and the CR. A p-value of <0.05 was defined as statistically significant.

## Results

The study population (300 patients) was divided into two groups according to their ingression in the CR program. Two hundred three patients participated in the CR program (CR group), and 97 patients did not (non-CR group). Demographic and clinical characteristics, as well as laboratory findings of the population, as listed in Table 1 . History of acute coronary syndrome, CABG, and PCI was similar in both groups ( Table 1 ).

**Table 1:** – Clinical and laboratory characteristics of CR and non-CR groups

Variables	non-CR group (n=97)	CR group (n=203)	p value
Age (year)	57.9±7.4	56.2±7.8	0.072
Gender (Male %)	74(76.3%)	159(78.3%)	0.399
Hypertension (n, %)	82(84.5%)	184(90.6%)	0.088
Diabetes mellitus (n, %)	20(20.6%)	55(27.1%)	0.142
Current smoker (n, %)	11(11.3%)	27(13.3%)	0.391
BMI (kg/m^2^)	28.2±3.1	28.3±3.9	0.885
PCI history (n, %)	61(62.9%)	117(57.6%)	0.230
ACS history (n, %)	28(28.9%)	50(24.6%)	0.259
CABG history (n, %)	19(19.6%)	37(18.2%)	0.446
LVEF %	57.1±5.3	58.3±5.6	0.086
**CAD severity**			
1 vessel	35(36.1%)	89(44.1%)	
2 vessels	58(59.8%)	98(48.5%)	
>2 vessels	4(4.1%)	15(7.4%)	0.155
Gensini Score	54.1±28	50.1±28.2	0.266
ASA (n, %)	95(97.9%)	200(98.5%)	0.745
P2Y12 inhibitors (n, %)	60(61.5%)	115(56.6%)	0.493
Beta-blockers (n, %)	82(84.5%)	158(77.8%)	0.113
Calcium-channel-blockers (n, %)	42(43.3%)	91(44.8%)	0.451
RAAS inhibitors (n, %)	80(82.5%)	165(81.3%)	0.469
Statin (n, %)	92(94.8%)	197(97.5%)	0.377
Fasting plasma glucose (pre) (mg/dL)	102.6±27.1	106.2±33.7	0.307
Creatinine (pre) (mg/dl)	0.9±0.21	0.87±0.21	0.374
Hemoglobin (pre) (g/dL)	13.4±1.02	13.6±1.07	0.135
MPV (pre)	8.7±0.98	9.1±1.02	0.010
MPV (post)	8.6±0.98	7.9±0.82	<0.001
ΔMPV*	-0.10 (-2.00-0.00)	-1.10(-1.40-[-0.90])	<0.001*
ΔMPV %*	-1.15 (-2.37-0.00)	-12.63(-14.46-[-10.11])	<0.001*
ΔLDL-c (mg/dL)*	-2.00 (-1.00-0.00)	-20.00 (-36.00-[-10.00])	<0.001*
ΔTG (mg/dL)*	-9.00 (-10.50-[-1.00])	-21.00 (-49.00-[-10.00])	<0.001*
ΔHDL-c (mg/dL)*	1.00 (0.00-1.00)	4.00 (3.00-6.00)	<0.001*
ΔWBC (10^3^/µ^3^)*	0.10 (-0.25-0.30)	-0.70(-1.20-[-0.35])	<0.001*
ΔNeutrophil (10^3^/µ^3^) *	-0.10 (-0.50-0.03)	-0.80 (-1.50-[-0.39])	<0.001*
ΔCRP (mg/dL) *	-0.10 (-0.10-0.10)	-0.80 (-1.80-[-0.40])	<0.001*

Data are presented as mean ± standard deviation or number of patients and percentage of patients. CR: cardiac rehabilitation; Δ: delta; BMI: body mass index; PCI: percutaneous coronary intervention; ACS; acute coronary syndrome; CABG: coronary artery bypass graft; LVEF: left ventricular ejection fraction; CAD: coronary artery disease; ASA: acetylsalicylic acid; RAAS: rennin-angiotensin-aldosterone; MPV: mean platelet volume; LDL: low density lipoprotein; TG: Triglycerides; HDL: high density lipoprotein; WBC: white blood cell; CRP: C-reactive protein; pre: before CR. *Comparison by Mann-Whitney U test at p<0.05. Values were described as medians with inter-quartile range (25^*th*^and 75^*th*^percentile).

The decrease in MPV was greater in the CR group than in the non-CR group [((-1.10(-1.40-(-0.90)) vs. (-0.10 (-2.00-0.00)) vs.; p<0.001, Table 1 ]. The correlation between ΔMPV and variables is shown in Table 2 . As shown in Figure 1 , the ΔMPV had a strong positive correlation with CR (r = 0.750, p < 0.001).

**Table 2:** – Correlation between ΔMPV and variables

	ΔMPV	
	r	p value
Δneutrophil	0.326	<0.001
ΔLDL-c	0.478	<0.001
ΔWBC	0.412	<0.001
ΔCRP	0.572	<0.001
ΔTG	0.439	<0.001
LVEF	-0.133	0.021
hemoglobin (pre)	-0.139	0.019
fasting plasma glucose (pre)	-0.129	0.026
ΔHDL-c	-0.537	<0.001
CR	0.750	<0.001
age	0.034	0.563
BMI	-0.022	0.727

Δ: delta; MPV: mean platelet volume; LDL: low density lipoprotein; TG: Triglycerides; LVEF: left ventricular ejection fraction; HDL: high density lipoprotein; WBC: white blood cell; CRP: C-reactive protein; CR: cardiac rehabilitation; BMI: body mass index; pre: before CR.

**Figure 1:** Correlation of ΔMPV with CR.

## Discussion

This study showed that exercise-based CR has a strong relationship with MPV reduction in patients with CAD.

Studies on the effect of exercise on platelet activation have yielded conflicting results so far. In agreement with our study, Yazıcıet al.^7^reported that a lifestyle change that includes at least 180 minutes/week of moderate-intensity physical activities decreased MPV in pre-hypertensive patients. Also, Rauramaa et al.^12^showed that low to moderate intensity exercise training was associated with diminished platelet aggregation. Contrary to these results, it was shown that exercise stress test increased MPV in patients with CAD.^11 , 13^Also, it has been shown that graded resistance exercises increase MPV.^12 , 14^

Exercise training can bring beneficial effects on platelets via different mechanisms. With exercise training, the pulsatile flow in the aorta increases, and this might induce an acute release and upregulation of nitric oxide (NO), which is a potent mediator of antiplatelet effects and suppression of platelet reactivity.^7 , 15^It is known that CR increases HDL cholesterol, which can stimulate platelet production and thereby decrease platelet activation.^9^In our study, ΔHDL-cholesterol was found to be independently correlated with a decrease in MPV.

MPV has been recognized as an inflammatory marker in cardiovascular, cerebrovascular, rheumatologic, and gastroenterological diseases.^16^Recently, it was shown that the presence of activated megakaryocytes in bone marrow correlates with increased circulating levels of IL-6 in patients with atherosclerosis.^17^Previous research provided evidence that a physically active lifestyle is associated with lower whole-body inflammatory biomarkers, and the anti-inflammatory actions of chronic exercise training are evident after as little as 2 to 12 weeks of supervised exercise training.^18^In our study, we demonstrated that ΔCRP was one of the independent predictors of ΔMPV. Reduction in MPV after CR might be explained by the anti-inflammatory effect of exercise-based CR programs.

The contradictions in the results of studies can be explained by various aspects of exercise affecting platelet functions such as different exercise intensity, duration, and various fitness levels of the subjects.^7^Acute intense exercise is proven to increase plasma pro-inflammatory cytokines.^18^MPV can reflect the changes in platelet stimulation and the rate of platelet production level.^13^An increase in MPV after exercise may be attributed to the fresh release of young large platelets, particularly from the splenic pool, into the circulation.^7^In this study, we implemented the CR program five days per week for a total of six weeks with a gradually increasing density, according to the individual’s exercise capacity.

Atherosclerosis itself can stimulate bone marrow megakaryocytes, which was shown to be associated with acute coronary syndrome, by causing circulating platelet consumption during athereogenesis.^17 - 19^One of the mechanisms of exercise-based CR potentially involved in the reduction of infarction and re-infarction may be related with reduction of MPV.^9 , 20^

Reticulated platelets are larger and possibly more active than non-reticulated platelets.^1^Moreover, reticulated or large platelets exhibit increased reactivity despite anti-platelet therapy.^21 , 22^Since it has been shown that high MPV is an independent risk factor for future myocardial infarction (MI) and recurrent MI, being associated with acute coronary syndrome or cardiovascular risk factors,^1 , 19 , 23^the questioning on how to decrease MPV has already been raised.^17 , 23^Although it was previously shown that statin treatment decreases MPV,^21^in our study most of the patients were already on statins, so we showed the additive effect of CR on MPV reduction.

### Study limitations

The present study has some limitations. First, we evaluated MPV only once and did not asses changes in it over time. Second, some anti-platelet drugs might affect platelet size.

## Conclusion

This study showed that exercise-based CR has a strong relationship with MPV reduction in patients with CAD. We consider that decreased platelet activation with exercise-based CR might play an important role in reducing thrombotic risk in patients with stable CAD.
